# Phosphorylation of *Arabidopsis* transketolase at Ser^428^ provides a potential paradigm for the metabolic control of chloroplast carbon metabolism

**DOI:** 10.1042/BJ20130631

**Published:** 2014-02-14

**Authors:** Agostinho G. Rocha, Norbert Mehlmer, Simon Stael, Andrea Mair, Nargis Parvin, Fatima Chigri, Markus Teige, Ute C. Vothknecht

**Affiliations:** *Department of Biology I, LMU Munich, Groβhaderner Str. 2-4, D-82152 Planegg-Martinsried, Germany; †Department of Biochemistry, MFPL, University of Vienna, Dr. Bohr Gasse 9/5, A-1030 Vienna, Austria; ‡Department of Molecular Systems Biology (MoSys), University of Vienna, Althanstr. 14, A-1090 Vienna, Austria; §Center for Integrated Protein Science (Munich), Department of Biology, LMU Munich, D-82152 Martinsried, Germany

**Keywords:** calcium signalling, Calvin–Benson–Bassham cycle, carbon metabolism, pentose phosphate pathway, phosphorylation, transketolase (TKL), CAS, calcium-sensing receptor, CBB, Calvin–Benson–Bassham, CDPK, calcium-dependent protein kinase, cpCKII, chloroplast casein kinase II, E4P, erythrose 4-phosphate, E4PDH, E4P dehydrogenase, FBPase, fructose 1,6-bisphosphate, F6P, fructose 6-phosphate, G3P, glyceraldehyde 3-phosphate, G3PDH, α-G3P-dehydrogenase, OPPP, oxidative pentose phosphate pathway, RBP, ribulose 1,5-bisphosphate, R5P, ribose 5-phosphate, S7P, sedoheptulose 7-phosphate, TKL, transketolase, TPI, triosephosphate isomerase, TPP, thiamine pyrophosphate, Var1, VARIEGATED 1, X5P, xylulose 5-phosphate

## Abstract

Calcium is an important second messenger in eukaryotic cells that regulates many different cellular processes. To elucidate calcium regulation in chloroplasts, we identified the targets of calcium-dependent phosphorylation within the stromal proteome. A 73 kDa protein was identified as one of the most dominant proteins undergoing phosphorylation in a calcium-dependent manner in the stromal extracts of both *Arabidopsis* and *Pisum*. It was identified as TKL (transketolase), an essential enzyme of both the Calvin–Benson–Bassham cycle and the oxidative pentose phosphate pathway. Calcium-dependent phosphorylation of both *Arabidopsis* isoforms (AtTKL1 and AtTKL2) could be confirmed *in vitro* using recombinant proteins. The phosphorylation is catalysed by a stroma-localized protein kinase, which cannot utilize GTP. Phosphorylation of AtTKL1, the dominant isoform in most tissues, occurs at a serine residue that is conserved in TKLs of vascular plants. By contrast, an aspartate residue is present in this position in cyanobacteria, algae and mosses. Characterization of a phosphomimetic mutant (S428D) indicated that Ser^428^ phosphorylation exerts significant effects on the enzyme's substrate saturation kinetics at specific physiological pH values. The results of the present study point to a role for TKL phosphorylation in the regulation of carbon allocation.

## Abstract 2

Stromal transketolase was identified as a target of calcium-dependent phosphorylation. The phosphorylated Ser^428^ is highly conserved in transketolases from vascular plants and affects the substrate saturation kinetics of the enzyme indicating a role for phosphorylation in the regulation of carbon allocation.

## INTRODUCTION

Chloroplast TKL (transketolase) is a key enzyme of plant carbon metabolism due to its amphibolic role in both the CBB (Calvin–Benson–Bassham) cycle and the OPPP (oxidative pentose phosphate pathway) [1,2]. During carbon fixation, TKL catalyses two reactions within the regenerative part of the CBB cycle, specifically the formation of X5P (xylulose 5-phosphate) and E4P (erythrose 4-phosphate) from F6P (fructose 6-phosphate) and G3P (glyceraldehyde 3-phosphate) as well as X5P and R5P (ribose 5-phosphate) from S7P (sedoheptulose 7-phosphate) and G3P [2]. Both reactions are principally reversible, but directionality is required to drive CO_2_ fixation by regeneration of RBP (ribulose 1,5-bisphosphate). Within the OPPP, the enzyme catalyses the same reactions, predominantly in the opposite direction to the CBB cycle [3]. The OPPP plays an important role in the production of NAD(P)H from glucose 6-phosphate in non-photosynthetic tissue and during the night. The pentose phosphates created can be utilized by other metabolic pathways, such as R5P for thiamine and nucleotide synthesis or E4P for the shikimate pathway, that produce aromatic amino acids as well as precursors for secondary metabolites involved in plant defence and signalling [4].

Although the CBB cycle is exclusively localized in the chloroplast, the precise cellular localization of all of the enzymatic steps of the OPPP is still a question of debate. A complete OPPP seems to exist in chloroplasts, but only a limited complement of the OPPP enzymes are present in the cytosol [5]. Consequently, translocators for pentose-phosphate intermediates of the OPPP have been identified in the chloroplast envelope [6]. The *Arabidopsis* genome contains two highly conserved paralogues of TKL (AtTKL1 and AtTKL2), both of which are predicted to reside in the chloroplast. Nevertheless, only AtTKL1 is ubiquitously expressed with the highest levels being in photosynthetic tissue. In contrast, AtTKL2 is expressed mainly during embryo development and is therefore unlikely to play an important role in carbon allocation in most tissues. An exclusive localization of TKL in the chloroplast raises the question of how enzyme activity is allocated between the different pathways and compartments. On the basis of immunogold EM, it has been suggested that there might be spatially separated centres for the CBB cycle and OPPP within the chloroplast [7]. Whether spatial distribution or directionality of the TKL reactions might be correlated with any kind of secondary modification of the protein is not known.

Regulation of cellular processes often occurs via protein phosphorylation, which has also been an early point of interest for photosynthetic research. Many thylakoid proteins undergo phosphorylation and several kinases involved in this regulation have been identified [8,9]. It was further suggested that phosphorylation cascades initiated at the thylakoid membrane may regulate chloroplast processes via soluble stromal kinases such as casein kinase II [10,11]. However, no direct evidence for the phosphorylation of chloroplast protein kinases by other kinases has so far been presented. Several large-scale phosphoproteomic studies have identified a wide range of phosphorylation targets in chloroplasts, including STN7 kinase, as potential substrates for phosphorylation [12,13]. All this information notwithstanding, the overall knowledge about chloroplast phosphorylation and the corresponding kinases and phosphatases remains scarce [14]. The phosphorylation reaction in turn can be regulated in different manners and in the cytosol the control by calcium via CDPKs (calcium-dependent protein kinases) is well described [15]. Calcium is an important second messenger and many environmental stimuli are transduced into an appropriate cellular response by transient changes in calcium concentration. However, calcium-dependent phosphorylation in chloroplasts has only been detected recently for three thylakoid proteins [16].

In the present study, we show that TKL is phosphorylated by stromal extracts in a calcium-dependent manner. Phosphorylation of TKL occurs at a serine/threonine residue that is conserved in the sequences of all TKL proteins from vascular plants, but is not found in TKLs from cyanobacteria, algae and mosses. Phosphorylation of TKL at this residue appears to differentially influence kinetic parameters at pH values representing light and dark conditions of stroma in photosynthetic tissue, indicating a role for TKL phosphorylation in the regulation of carbon allocation.

## MATERIALS AND METHODS

### Materials

Recombinant proteins were produced using the pTWIN (New England Biolabs) or pET21b (Invitrogen) system. A clone for the *Chlamydomonas reinhardtii* TKL (CrTKL; clone CL54b03) was obtained from the Kazusa DNA Research Institute (Chiba, Japan). For activity measurements, the following materials were purchased from Sigma: X5P, R5P, F6P, G3P, G3PDH-TPI (α-glyceraldehyde-3-phosphate dehydrogenase-triosephosphate isomerase) from rabbit muscle, β-NAD, β-NADH and TPP (thiamine pyrophosphate). Radioactive [γ-^32^P]ATP (111 TBq/mmol) and [γ-^32^P]GTP (222 TBq/mmol) was purchased from PerkinElmer. The primary polyclonal antibody (anti-AtTKL1) was generated in the rabbit (Biogenes) and raised against purified recombinant mature AtTKL1 protein. Antibodies against cytosolic UGPase (UDP-glucose pyrophosphorylase) and mitochondrial AOX1/2 (alternative oxidase 1/2) were purchased from Agrisera. Antibodies against stromal FBPase (fructose 1,6-bisphosphatase) and mitochondrial VDAC (voltage-dependent anion channel) were a gift from Dr J. Soll (LMU Munich, Munich, Germany).

### Expression and purification of recombinant proteins

AtTKL1 (At3g60750) and AtTKL2 (At2g45290) lacking the N-terminal 67 amino acids (i.e. the chloroplast-targeting peptide), as well as the AtTKL1_S428A_ and AtTKL1_S428D_ with point mutations at Ser^428^, were cloned into pTWIN1 in-frame with the N-terminal intein tag. All AtTKL constructs were expressed in *Escherichia coli* strain ER2566 cells and purified under native conditions using the IMPACT™-TWIN system (New England Biolabs) and the intein tag was cleaved during affinity purification following the manufacturer's instructions. CrTKL and E4PDH (E4P dehydrogenase) from *E. coli* were cloned into pET21b in-frame with a C-terminal His_6_ tag, expressed in *E. coli* strain BL21 cells and purified under native conditions using Ni-NTA (Ni^2+^-nitrilotriacetate) resin according to standard protocols. The mature form of the *Arabidopsis* cpCKII (chloroplast casein kinase II; At2g23070) lacking the N-terminal 85 amino acids was cloned as an ApaI–NotI fragment into the pGEX4-T1 expression vector and purified as an N-terminal GST-fusion protein. The prediction of the mature form was on the basis of a sequence alignment with mature cpCKII from *Sinapis alba* as described in [17]. A list of all primers used for cloning can be found in Supplementary Table S1 (at http://www.biochemj.org/bj/458/bj4580313add.htm).

### Plant growth and preparation of chloroplast proteins

*Arabidopsis thaliana* (cultivar Columbia Col-0) was grown in soil at 18–22°C with an 8 h/16 h light/dark period at 150 μmol/m^2^ per s and *Pisum sativum* (cultivar Arvika) on vermiculite with a 16 h/8 h light/dark period at 250 μmol/m^2^ per s. Chloroplasts were purified from 6–7-week-old *Arabidopsis* leaves (~500 g of fresh mass) as described in [18] and from 7–9-day-old pea leaves (~50 g of fresh mass) as described in [19]. In both cases, leaves were harvested at the end of the dark period. Chloroplasts were disrupted by suspension in lysis buffer [20 mM Tricine (pH 7.6), 10% (v/v) glycerol and 1 mM DTT] supplemented with protease inhibitors (Complete™ EDTA free; Roche), phosphatase inhibitors (Phospho-Stop; Roche) and 5 mM EGTA. After incubation on ice for 15 min, membranes and soluble components were separated by centrifugation at 60000 ***g*** for 10 min. To extract the membrane-associated proteins, the membrane pellet was subsequently resuspended in lysis buffer containing 0.8 M NaCl and centrifuged again at 60000 ***g*** for 10 min. Supernatants from the first and second centrifugation were combined, concentrated and desalted into lysis buffer using Vivaspin™ 500 columns (GE Healthcare) and are referred to as the stromal protein fraction. The remaining pellet contained the membrane protein fraction. *C. reinhardtii* (cw15 cell wall-less strain) were grown as described previously [20] under illumination at 20 μmol/m^2^ per s at 25°C. Total cell extract was prepared as described above for chloroplasts. If not otherwise stated, all procedures were carried out at 4°C.

### Protein phosphorylation assays

Phosphorylation assays for the detection of *in vitro*-phosphorylated proteins were conducted using 10–20 μg of soluble stromal proteins from either *Arabidopsis* or *Pisum*. Assays were carried out for 25 min at room temperature (22°C) in a total volume of 50 μl in kinase buffer containing 20 mM Tricine (pH 7.6), 10 mM MgCl_2_, 10% (v/v) glycerol, 1 mM DTT, 5 μM ATP and 70–180 kBq of [γ^32^-P]ATP or [γ^32^-P]GTP. Depending on the experiment, assays were also supplemented with 5 mM CaCl_2_, 5 mM cadmium acetate, 5 mM zinc acetate, 5 mM CuCl_2_, 5 mM MnCl_2_, 5 mM NiSO_4_ or 2 mM EGTA. When recombinant TKL variants (100–200 ng) were used as a substrate, assays were carried out with catalytic amounts (50–100 ng) of stromal proteins, chloroplast membrane proteins or *Chlamydomonas* total cell extract. Proteins were separated by SDS gel electrophoresis and stained with Coomassie Brilliant Blue R-250. Radiolabelled proteins were detected by exposure to phosphoimager screens analysed on a Typhoon Trio imager (GE Healthcare) or by exposure to X-ray film (Fuji) at −80°C. For comparative quantification of labelling, the proteins were excised from the SDS gel and the incorporated γ-^32^P was measured by liquid scintillation counting.

### 2D PAGE separation

Chloroform/methanol-precipitated protein pellets from phosphorylation reactions were solubilized in rehydration buffer [7 M urea, 2 M thiourea, 2% (w/v) CHAPS, 0.5% IPG buffer, 0.002% Bromophenol Blue and 1 mM DTT]. Proteins were applied to 11 cm immobilized DryStrip gels (pH 3–8; non-linear; GE Healthcare) and separated using the Bio-Rad Laboratories Protean IEF Cell Isoelectric Focusing system following the manufacturer's instructions. Afterwards, the strips were equilibrated consecutively in equilibration buffer [75 mM Tris/HCl (pH 8.8), 6 M urea, 30% (v/v) glycerol, 2% (w/v) SDS and 0.002% Bromophenol Blue] containing 10 mg/ml DTT and in equilibration buffer containing 25 mg/ml iodacetamide for 20 min each. Proteins were separated in the second dimension by SDS/PAGE (10% gel) and analysed as described above.

### Immunopurification of TKL after the protein phosphorylation assay

Using stromal extracts from *Arabidopsis* or *Pisum*, phosphorylation assays were conducted with approximately 100 μg of total protein in 200 μl of kinase buffer supplemented with 5 mM CaCl_2_. For the purpose of LC–MS/MS analysis, the same assay was conducted in parallel, but without radiolabelled [γ-^32^P]ATP. Reactions were carried out for 25 min at room temperature and subsequently incubated for 1 h with 10 μl of Protein A–Sepharose and 6 μl of anti-AtTKL1 antiserum. The beads were washed three times with 800 μl of 20 mM Tricine/NaOH (pH 7.6) and 1 mM DTT. Bound proteins were eluted with 50 μl of SDS solubilization buffer and the samples were analysed by SDS/PAGE (12% gel) separation and protein staining with Coomassie Brilliant Blue R-250. The incorporation of radioactivity was measured by exposing the dry gel to X-ray film at −80°C.

### Protein identification by MS

MS/MS analysis and data interpretation were carried out as described in [16]. For the identification of phosphopeptides, spectra were reanalysed with Proteome Discoverer 1.2 (Thermo Scientific). The Mascot search was performed as described in [20a]. Results were prefiltered using XCorr (+2, +3, +4)=2, 2.5, 3. Manual validation of the identified phosphopeptide included comparison with the fragmentation pattern of the unphosphorylated counterpart and comparison of the relative retention to the unphosphorylated counterpart. Site localization was checked by manual inspection at the spectrum level.

### TKL activity assay

Conversion of X5P and R5P into G3P and S7P was measured as described previously in [21] with minor changes. The 200 μl reaction mixture contained 20 mM glycylglycine/NaOH (pH 7.2 or 8.0), 0.1 mM TPP, 0.14 mM β-NADH, 15 mM MgCl_2_, 5 mM CaCl_2_, 20 units of rabbit muscle G3PDH-TPI (based on TPI units) and 0.1 μg of recombinant TKL. To measure the kinetic parameters for X5P, the reactions contained 1.7 mM R5P and the X5P content varied between 0.1 and 3.0 mM. To measure the kinetic parameters for R5P, the reactions contained 1.5 mM X5P and the R5P content varied between 0.125 and 3.0 mM.

Conversion of F6P and G3P into E4P and X5P was measured as described in [22] with minor changes. The 200 μl reaction mixture contained 50 mM Tris/HCl (pH 7.2 or 8.0), 0.1 mM TPP, 2.5 mM β-NAD, 15 mM MgCl_2_, 5 mM CaCl_2_, 10 μg of E4PDH and 2–3 μg of recombinant TKL. To measure the kinetic parameters for F6P, the reactions contained 2 mM G3P and the F6P content varied between 0.1 and 20 mM. To measure the kinetic parameters for G3P, the reactions contained 5 mM F6P and the G3P content varied between 0.125 and 2.0 mM. Conversion of NADH into NAD or vice versa was measured as the change in *A*_340_ (ε=6220 M^−1^·cm^−1^) using a Tecan Safire2 microplate reader at 30°C. Specific activity was expressed as μmol of oxidized/reduced NADH/min per mg of TKL. Kinetic parameters were determined using the Michaelis–Menten equation *V*=*V*_max_×[*S*]/(*K*_m_ + [*S*]), where *V* is the rate of NADH formation/depletion and [*S*] is the concentration of the various sugars. Non-linear regression analysis (*n*=4) was performed in GraphPad Prism Version 5.01 for Windows.

### Transient expression in tobacco leaves

AtTKL1–YFP and AtTKL2–YFP were transiently expressed in tobacco leaves as described in [23]. Fluorescence images were obtained using the confocal laser-scanning microscope TCS-SP5 (Leica Microsystems) and the Leica LAS AF software.

### Bioinformatic analyses

Sequence alignments were obtained using ClustalX 2.0 [24] and box-shading was performed by BOXSHADE 3.31 (http://www.ch.embnet.org/software/BOX_form.html). The logo representing the residue probability around the phosphorylation site was generated using the Weblogo 3 program [25]. A complete list of the accession numbers is given in Supplementary Table S2 (at http://www.biochemj.org/bj/458/bj4580313add.htm). The TKL dimer structure was taken from the 3D structure of the maize TKL (PDB code 1ITZ). Ser^428^ was manually highlighted using UCSF Chimera [26].

## RESULTS AND DISCUSSION

### Identification of stromal targets of calcium-dependent phosphorylation

**Figure 1:**
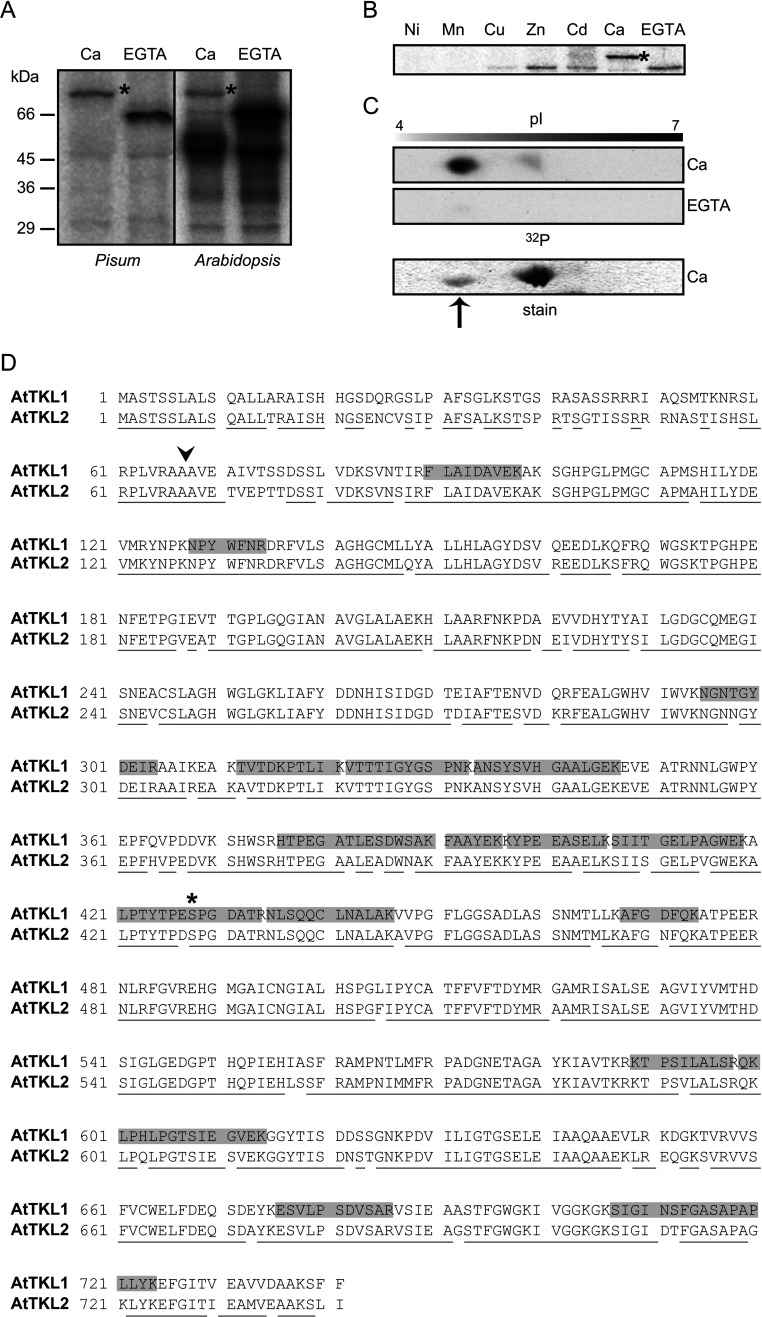
Calcium-dependent phosphorylation of stromal proteins (**A**) Autoradiograms of SDS/PAGE-separated stromal proteins after phosphorylation assays in the presence of either calcium or EGTA showed a 73 kDa protein that was phosphorylated only in the presence of calcium (marked by an asterisk) in both *Pisum* (15 μg of total protein/lane) and *Arabidopsis* (10 μg of total protein/lane). Molecular mass is given on the left-hand side in kDa. (**B**) Phosphorylation assays with *Arabidopsis* stromal proteins (10 μg of total protein/lane) performed in the presence of different cations or EGTA showed a strict calcium dependency for the phosphorylation of the 73 kDa protein (marked by an asterisk). (**C**) Autoradiogram of stromal protein (250 μg) from *Arabidopsis* separated by 2D PAGE (isoelectric focusing followed by SDS/PAGE) after phosphorylation assays in the presence of calcium or EGTA. Only the area around 70 kDa is shown (for the whole gels see Supplementary Figure S3 at http://www.biochemj.org/bj/458/bj4580313add.htm). The 73 kDa protein indicated by an arrow (lower panel) was excised from a Coomassie Blue-stained gel and analysed by MS/MS. (**D**) Amino acid sequence alignment of the two *Arabidopsis* TKL isoforms AtTKL1 and AtTKL2. Grey boxes indicate peptides found by MS/MS (Supplementary Table S3 at http://www.biochemj.org/bj/458/bj4580313add.htm). The arrowhead indicates the potential cleavage site for the chloroplast-targeting sequence as predicted by similarity to TKL from spinach [7]. The asterisk indicates the identified phosphoserine. Lines underneath the sequence alignment indicate amino acid conservation.

The aim of the present study was to identify novel chloroplast targets of calcium-dependent phosphorylation using stromal extracts from either *Pisum* or *Arabidopsis* leaves. To ensure that any observed phosphorylation was not caused by contamination from other cellular compartments, we verified the purity of the stroma extracts by Western blot analysis (Supplementary Figure S1 at http://www.biochemj.org/bj/458/bj4580313add.htm). We then used the stromal extracts to perform phosphorylation assays with [γ-^32^P]ATP in the presence of calcium or EGTA (Figure 1A and Supplementary Figure S2 at http://www.biochemj.org/bj/458/bj4580313add.htm). In general, most of the phosphorylated proteins showed no changes in the absence or presence of calcium. A 73 kDa protein was phosphorylated only in the presence of calcium in both *Pisum* and *Arabidopsis* stroma (Figure 1A, asterisk), whereas a phosphorylated protein of approximately 50 kDa was only seen in *Arabidopsis*. Owing to its calcium-dependent phosphorylation in both *Pisum* and *Arabidopsis*, we investigated further the 73 kDa protein.

To ensure calcium specificity, we next performed the phosphorylation reaction in the presence of different divalent cations (Figure 1B). The 73 kDa protein was phosphorylated only in the presence of calcium; in contrast, a 65 kDa protein that was not phosphorylated in the presence of calcium (compare Figures 1A and 1B) was phosphorylated in the presence of several, but not all, divalent cations, indicating that the phosphorylation of this protein was not correlated with the presence or absence of calcium.

We then separated *Arabidopsis* samples using 2D PAGE by isoelectric focusing in the first and SDS/PAGE in the second dimension (Figure 1C and Supplementary Figure S3 at http://www.biochemj.org/bj/458/bj4580313add.htm). A protein matching the radioactive spot at 73 kDa could confidently be identified on the corresponding Coomassie Brilliant Blue-stained gels (Figure 1C, compare ^32^P and stain). The protein was analysed by MS/MS and several peptide masses could be matched to the predicted protein sequence of TKL from *Arabidopsis* (Figure 1D, grey boxes). TKL was also identified in MS/MS analyses of both *Pisum* and *Arabidopsis* when samples were analysed after 1D SDS/PAGE separation (results not shown). Three peptides matched the coding sequence of both *Arabidopsis* TKLs and six peptides were specific for AtTKL1, the major isoform of TKL in *Arabidopsis* leaf tissue [27], whereas no AtTKL2-specific peptide could be found. We thus conclude that AtTKL1 is the protein identified by MS/MS. Mature AtTKL1, after cleavage of the targeting peptide (Figure 1D, arrowhead), has a predicted protein mass of 73 kDa and a pI of 5.33, which both correlate well with the features of the 73 kDa phosphoprotein observed upon 1D and 2D PAGE separation.

### A stromal protein kinase phosphorylates TKL in a calcium-dependent manner

**Figure 2:**
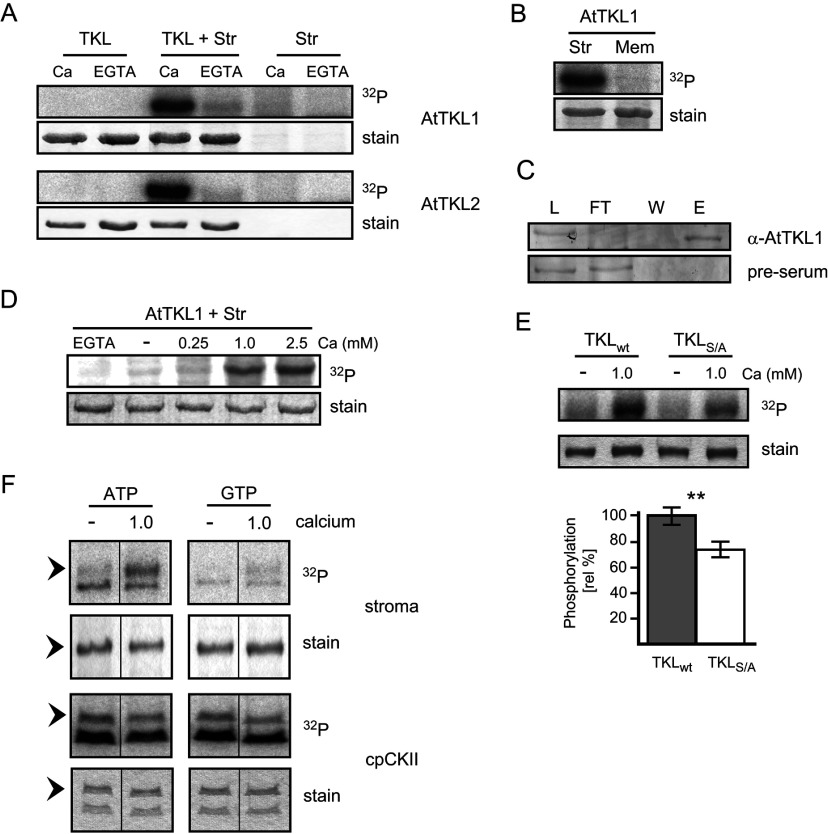
TKL is phosphorylated by a stromal kinase (**A**) Purified recombinant AtTKL1 and AtTKL2 (1 μg/assay) can be phosphorylation by ‘catalytic’ amounts (0.1 μg of total protein/assay) of stromal extract (Str) only in the presence of calcium. Purified TKL and stromal extract alone were used as controls. (**B**) AtTKL1is phosphorylated by a stromal- (Str) and not a membrane- (Mem) localized kinase. (**C**) Immunopurification using stromal proteins from *Arabidopsis* (8 μg per assay) with an antiserum raised against recombinant AtTKL1 (α-AtTKL1). Pre-immune serum was used as a control for the specificity of the antibody (pre-serum). L, load; FT, flow-through; W, wash; E, eluate. (**D**) Phosphorylation of purified recombinant AtTKL1 by stromal extracts in the presence of EGTA (EGTA), neither calcium nor EGTA (-), and different calcium concentrations (0.25, 1.0 and 2.5). (**E**) Phosphorylation of recombinant wild-type AtTKL (TKL_wt_) and the AtTKL_S428A_ variant (TKL_S/A_) in the absence and presence of calcium shows a significant reduction in phosphorylation of the mutant variant (upper panel). The difference in incorporated radioactivity was quantified by liquid scintillation and each value represents the mean ± S.D. from four independent experiments (lower panel). Significance was calculated using a standard Student's *t* test (**P<0.01). (**F**) Calcium-dependent phosphorylation of AtTKL1 by stromal extract (stroma) requires ATP and calcium. In contrast, recombinant cpCKII phosphorylates AtTKL1 independently of calcium and can also utilize GTP. TKL is marked by arrowheads since the slightly smaller cpCKII is autophosphorylated in these assays. In (**A**), (**B**), (**D**) and (**F**), both the autoradiograms (^32^P) as well as the Coomassie Blue-stained gels (stain) are shown.

To confirm the calcium-dependent phosphorylation of TKL, we performed assays with purified recombinant AtTKL1 and AtTKL2 using low amounts of *Arabidopsis* stromal extract to ensure that the endogenous 73 kDa protein would not interfere with the results. Assays with recombinant AtTKL1 or AtTKL2 alone showed no phosphorylation (Figure 2A, TKL). Similarly, only a very weak signal at 73 kDa could be observed in the stromal extract in the presence of calcium (Figure 2A, Str). In the presence of both stromal extract and recombinant protein, a strong phosphorylation of recombinant AtTKL1 and AtTKL2 could be observed solely in the presence of calcium (Figure 2A, TKL + Str), thereby confirming the identity of the 73 kDa calcium-dependent phosphoprotein as chloroplast TKL. Further experiments were then performed exclusively with AtTKL1, the dominant isoform in leaf tissue. To confirm the exclusive localization of the corresponding protein kinase in the chloroplast stroma, we separated chloroplast proteins into soluble and membrane-bound fractions and performed phosphorylation assays using both fractions (Figure 2B). Recombinant AtTKL1 was only phosphorylated in the presence of soluble proteins, but not in the presence of membrane proteins. These results indicate that the corresponding kinase is a soluble stromal protein and not associated with the thylakoid membrane.

TKL represents the first stromal target of calcium-dependent phosphorylation. Indeed, the occurrence of calcium-dependent phosphorylation in chloroplasts was only shown recently for three thylakoid proteins, namely CAS (calcium-sensing receptor), Var1 (VARIEGATED 1) and PsaN [16]. In the present study, TKL phosphorylation occurs by a stroma-localized kinase, since no membrane proteins are required in this reaction. Comparably, the two thylakoid targets CAS and Var1 were shown to be phosphorylated on stroma-exposed domains by a high-salt-washed stromal extract [16] and it is thus possible that one and the same stromal kinase phosphorylates these proteins as well as TKL.

To better understand the calcium requirement of the TKL phosphorylation reaction, we compared the phosphorylation efficiency under different calcium concentrations (Figure 2D). Hardly any phosphorylation could be observed either in the presence of EGTA or the absence of added calcium. Phosphorylation could be observed when 250 μM calcium was included in the assay with stronger phosphorylation observed at 1 mM. Further increase of the calcium concentration to 2.5 mM did not yield stronger phosphorylation. These calcium concentrations are higher than what would be expected for high-affinity calcium-binding proteins, such as classical CDPKs, and are rather in line with the low affinity of calcium-binding proteins, such as the chloroplast calcium-binding protein CAS [28]. This could indicate that the kinase is activated by calcium binding to a low-affinity calcium-binding site within the protein itself or as part of a kinase-activating step. Alternatively, calcium might induce a conformational change of the target protein, i.e. TKL, as a prerequisite for efficient phosphorylation.

### AtTKL1 is phosphorylated at a conserved serine residue

To identify the position(s) at which TKL is phosphorylated, we performed a ‘cold’ (non-radioactive) calcium-dependent phosphorylation assay with *Arabidopsis* stromal proteins followed by immunoprecipitation using an antibody raised against recombinant AtTKL1 (Figure 2C and Supplementary Figure S4 at http://www.biochemj.org/bj/458/bj4580313add.htm). In the elution fraction a protein band of the correct molecular mass could be identified after SDS/PAGE separation (Figure 2C, α-AtTKL1). No protein could be precipitated with the pre-immune serum demonstrating the specificity of the antibody (Figure 2C, pre-serum). The immunoprecipitated protein was analysed by MS/MS with a special focus on the identification of phosphopeptides. The analysis confirmed the identity of the protein as AtTKL1. It also yielded a single phosphopeptide at Ser^428^ in the AtTKL1 sequence (Figure 1D and Supplementary Figure S5 at http://www.biochemj.org/bj/458/bj4580313add.htm). TKL had been identified previously as a phosphoprotein by several phosphoproteomic analyses of chloroplast proteins and they all identified the same phosphopeptide that we found in the present study [12,13,29,30]. They used proteins isolated directly from biological material without any additional phosphorylation reactions thereby confirming the *in vivo* phosphorylation of TKL at Ser^428^. The phosphoserine residue is conserved between AtTKL1 and AtTKL2; however, it should be noted that the identified phosphopeptide is specific for AtTKL1 since AtTKL2 contains an aspartate residue at position 427 just before the serine and not a glutamate (Figure 1D).

To confirm whether Ser^428^ is phosphorylated in a calcium-dependent manner, we constructed an AtTKL1 variant where this residue was mutated to alanine (AtTKL_S428A_). We then used both variants in phosphorylation assays with *Arabidopsis* stromal extracts (Figure 2E). As with the wild-type variant, no phosphorylation of AtTKL_S428A_ was observed in the absence of calcium. In the presence of calcium some phosphorylation of AtTKL_S/A_ was observed, but it was significantly less than the wild-type variant (Figure 2E). These results strongly support Ser^428^ as one site of calcium-dependent phosphorylation of AtTKL1. Quantification of radioactivity incorporation into the wild-type and mutant variant by liquid scintillation counting also indicated that there are other phosphorylation sites (Figure 2E) that were not detected by our phosphopeptide analysis due to the lack of complete peptide coverage (Supplementary Table S3 at http://www.biochemj.org/bj/458/bj4580313add.htm).

### Potential phosphorylation of AtTKL by cpCKII

Very little is known about soluble protein kinases in chloroplasts [14]. The most probable candidate for phosphorylation of TKL would appear to be a CDPK, but the phosphorylation site of TKL (PE[pS]P) shares no similarity with the recognition motif of plant CDPKs in agreement with the fact that the calcium requirement for TKL phosphorylation was rather high and no CDPKs have so far been described in chloroplasts [14]. Indeed, a large-scale analysis of chloroplast protein phosphorylation sites has identified a significant enrichment of casein kinase II and proline-directed kinase motifs [13]. The PE[pS]P motif around Ser^428^ is most reminiscent of the serine/proline phosphorylation motif of proline-directed kinases, but members of this kinase family have not been identified in chloroplasts and no direct calcium regulation of proline-directed kinases has been described. However, casein kinase II is known to have a preference for acidic amino acids in the phosphorylation site and it was suggested that cpCKII is mainly responsible for phosphorylation of stromal proteins [10,13]. Casein kinase II is also known to use ATP and GTP as co-substrates in its reaction [31] and it has been shown recently that the majority of phosphorylation events in the chloroplast stroma fall into this category [14]. We therefore analysed whether TKL can be phosphorylated with stromal extracts using GTP instead of ATP (Figure 2F, stroma). Hardly any TKL phosphorylation was observed with GTP either in the absence or the presence of calcium indicating that the respective stromal kinase was not able to utilize GTP. We also expressed *Arabidopsis* cpCKII in *E. coli* cells and used the purified protein in phosphorylation assays (Figure 2F, cpCKII). Predominately visible in the autoradiogram is the autophosphorylation of the kinase. In these *in vitro* assays, cpCKII was able to phosphorylate recombinant AtTKL1 (Figure 2F, arrowheads); however, contrary to the results with the stromal extract, recombinant cpCKII was able to utilize GTP and the phosphorylation reaction was not calcium-dependent. Therefore it appears unlikely that cpCKII is the kinase that phosphorylates AtTKL *in vivo*, but it cannot be fully excluded until the respective kinase is clearly identified.

### Phylogenetic distribution of TKL phosphorylation

**Figure 3:**
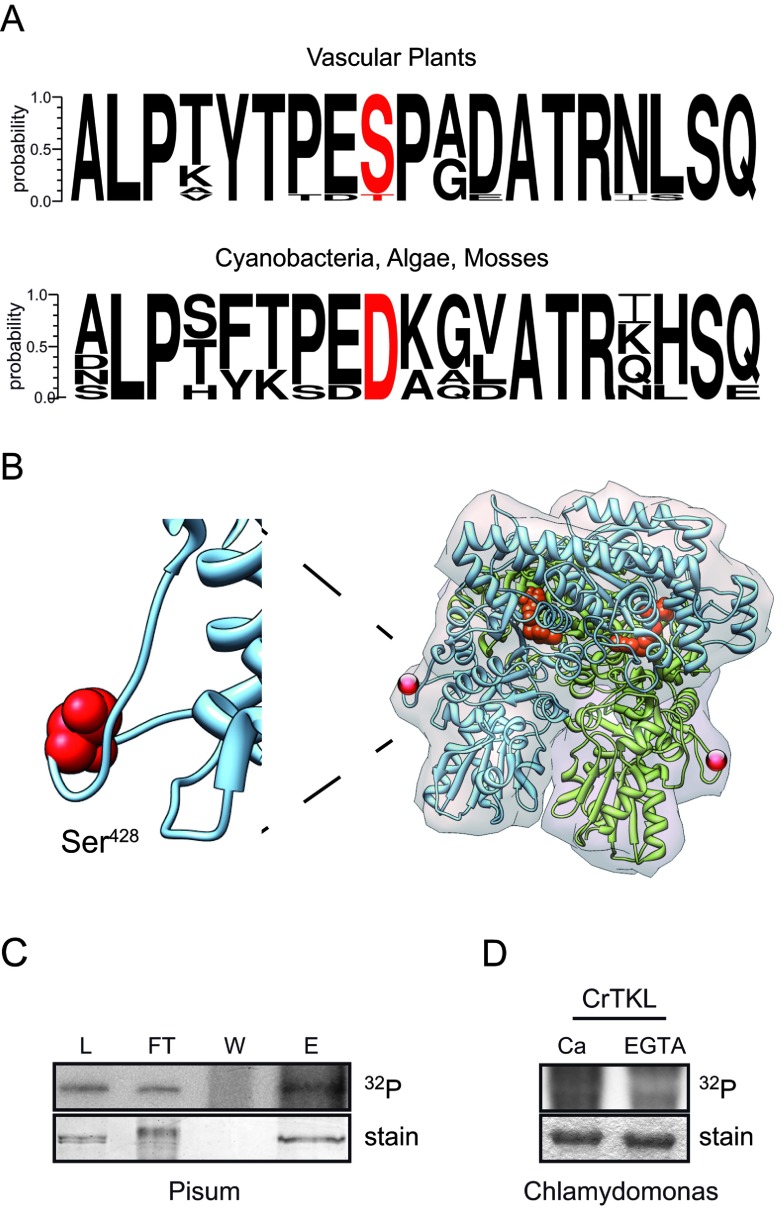
Phylogenetic conservation of TKL phosphorylation (**A**) Probability analysis showing that a serine (or very rarely threonine) residue is present at the position of the phosphoserine from AtTKL1 in TKLs from all vascular plants. TKLs from cyanobacteria, algae and mosses contain an aspartate residue in the same position. The analysis is based on the amino acid sequence alignment shown in Supplementary Figure S6 (at http://www.biochemj.org/bj/458/bj4580313add.htm). (**B**) The phosphorylation site (red sphere) was modelled on to the 3D structure of the maize TKL (PDB code 1ITZ), which contains a conserved serine residue in the same position as AtTKL1. The TPP cofactor is marked in orange. (**C**) Immunopurification using stromal protein from *Pisum* (10 μg) with the anti-TKL1 antiserum after a phosphorylation assay. The 73 kDa stromal phosphoprotein from *Pisum* can be immunopurified with the antiserum against AtTKL1. L, load; FT, flow-through; W, wash; E, eluate. (**D**) Recombinant TKL from *C. reinhardtii* (crTKL; 1 μg) is not phosphorylated by soluble protein extracts from the same organism.

A sequence alignment of different TKLs shows that the region around Ser^428^ is strongly conserved in photosynthetic organisms (Supplementary Figure S6 at http://www.biochemj.org/bj/458/bj4580313add.htm). An amino acid distribution analysis of this domain showed further that a serine (or very rarely threonine) residue is present at this position in all vascular plants (Figure 3A, upper panel) as well as in TKL from *Selaginella*. We thus wanted to confirm the phosphorylation of TKL from *Pisum* (PsTKL). *Pisum* stroma was used for a phosphorylation assay with [γ-^32^P]ATP and subsequently incubated with the anti-AtTKL1 antiserum. A protein with the correct molecular mass was immunopurified and the autoradiogram showed that this protein was radiolabelled (Figure 3C). Together with the results from Figure 1(A) this strongly supports that PsTKL can also be phosphorylated in a calcium-dependent manner.

TKLs from mosses, algae and cyanobacteria all possess aspartate in place of the phosphoserine even though the adjacent residues are quite conserved (Figure 3A, lower panel). Aspartate residues are not commonly phosphorylated in chloroplasts, making it unlikely that non-vascular plant TKLs are phosphorylated at this position. We nevertheless performed phosphorylation assays with recombinant TKL from *C. reinhardtii* (CrTKL) using soluble extracts from this alga (Figure 3D). No phosphorylation of the recombinant protein could be observed either in the presence or absence of calcium, suggesting strongly that CrTKL is not phosphorylated in the same manner as AtTKL or PsTKL; however, aspartate can mimic phosphorylated serine due to similarity in charge and shape [32]. Comparative genomic analysis has revealed that a replacement of acidic residues by a phosphorylatable serine or threonine residue has occurred frequently during evolution [33] and TKL seems to represent another such example. A serine residue is present in TKL of *Selaginella*, the only lycophyte sequence so far available. Phylogenetically, lycophytes are placed in between the bryophytes and the euphyllophytes, and they have been suggested as key models for the understanding of major evolutionary adaptation to life on land, such as vascular tissue, leaves, stems and lignification [34]. Phosphorylation of TKL from the lycophytes onwards might thus indicate a role for this process in environmental adaptation, including abiotic stresses, such as drought, or biotic stresses, such as pathogen attack.

### Influence of phosphorylation on TKL activity

TKLs from vascular plants are known to form functional dimers with the active site located in a groove formed by the contact site of the two monomers [35]. On the basis of a 3D model of the crystalized maize TKL, Ser^428^ is localized between α-helix 12 and β-sheet 6 just at the beginning of the so-called central domain. Although the central domain is involved in dimer interface formation, the phosphoserine is located in a loop that extends to both sides out of the compact centre of the dimer (Figure 3B). This placement does not lend itself to assume a direct influence of phosphorylation on TKL homodimerization and we could not observe any difference in oligomerization status either by Blue native PAGE or size-exclusion chromatography (results not shown).

**Figure 4:**
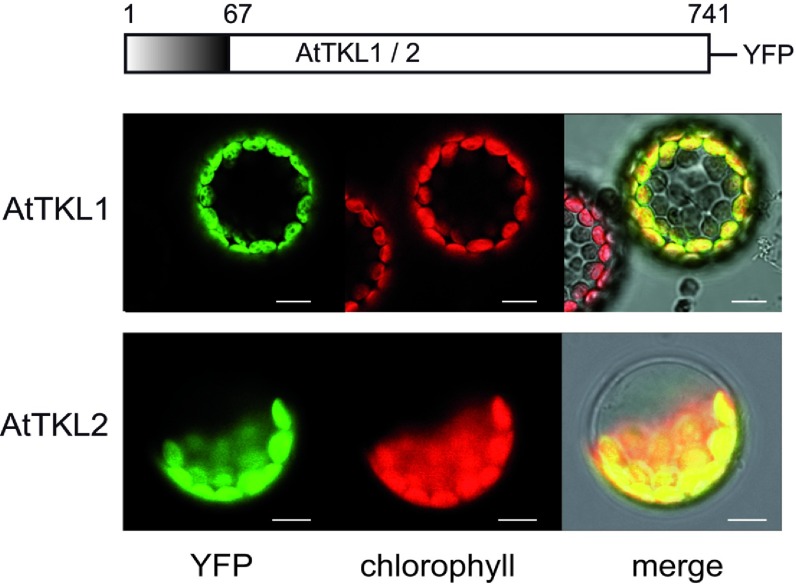
TKL is localized exclusively in chloroplasts AtTKL1 and AtTKL2 were transiently expressed as YFP-fusion proteins in tobacco leaf cells. Fluorescence of YFP (green) and chlorophyll (red) was analysed by laser-scanning fluorescence microscopy using protoplasts isolated from transformed leaves. An overlay of both fluorescence signals with the bright-field picture (merge) shows the exclusive localization of both proteins in chloroplasts. Scale bars, 10 μm.

One of the greatest enigmas with regard to TKL is its functional distribution between different pathways that allegedly take place in the same compartment and that utilize the opposite directions of two readily reversible reactions. However, the exact distribution of the components of the OPPP is still a matter of debate. It was suggested that plant TKL is present exclusively in chloroplasts even though several steps of the OPPP are localized in the cytosol. We therefore re-assessed the subcellular localization of both AtTKL1 and AtTKL2 by fluorescence microscopy using transient expression of YFP-fusion proteins in tobacco leaf cells. In both cases, the YFP signal showed a clear overlap with the chlorophyll fluorescence (Figure 4), with no signal visible anywhere else in the cell, thereby strongly supporting an exclusive localization of both TKLs in chloroplasts. This necessitates that the same enzyme is involved in both pathways.

**Figure 5:**
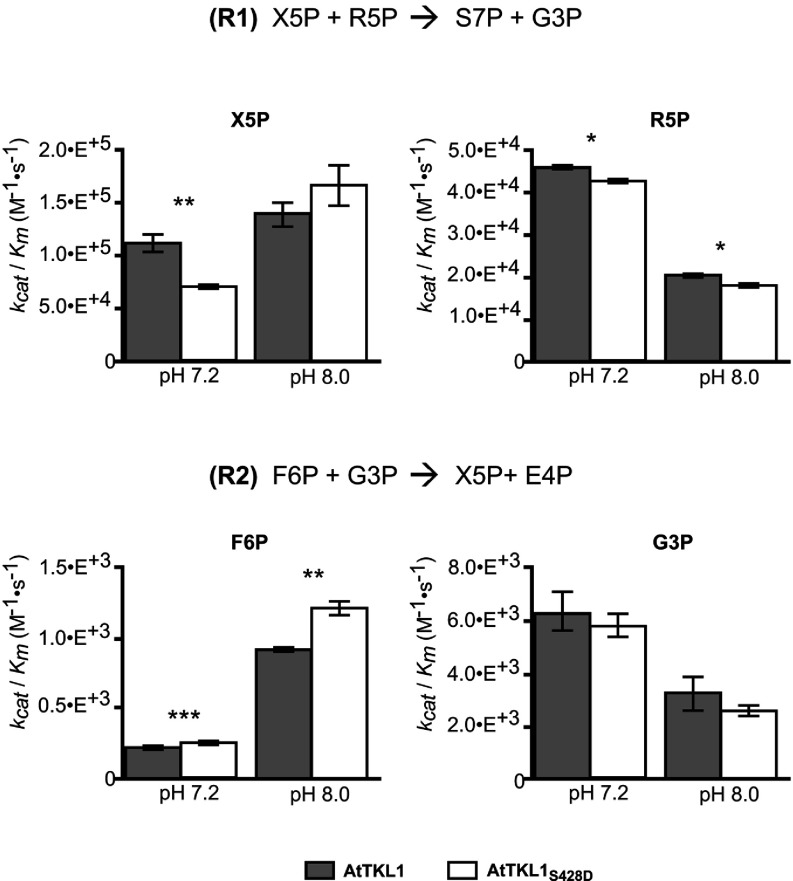
Catalytic efficiency of AtTKL1 and AtTKL1_S428D_ Enzymatic reactions were measured either with X5P and R5P or with F6P and G3P at pH 7.2 and 8.0 as described in the Materials and methods section. The *k*_cat_*/K*_m_ value was calculated for all sugars and each value represents the mean±S.D. for four to five independent determinations. Significance of the parameters was calculated using a standard Student's *t* test (*P<0.05, **P<0.01 and ***P<0.001). E^+3^=×10^3^ etc.

We thus wanted to elucidate a potential influence of Ser^428^ phosphorylation on enzyme activity using non-phosphorylated wild-type AtTKL1 compared with a phosphomimetic mutant containing a serine–aspartate mutation at position 428 (AtTKL1_S428D_). Unfortunately, not all reactions of TKL can be measured *in vitro* due to a lack of availability of substrates and/or a suitable assay. We thus analysed the formation of S7P and G3P from X5P and R5P as well as the formation of X5P and E4P from F6P and G3P (Figure 5 and Supplementary Table S4 at http://www.biochemj.org/bj/458/bj4580313add.htm). We also measured the enzyme activity at two different physiological pH values (7.2 and 8.0) representing dark and light conditions of stroma.

The kinetic parameters of AtTKL1 for the OPPP reaction X5P+R5P→S7P+G3P (R1) were assessed using a coupled reaction with TPI and G3PDH. The CBB reaction F6P+G3P→E4P+X5P (R2) was measured by a coupled reaction using E4PDH. All reactions followed Michaelis–Menten kinetics and means±S.D. for *V*_max_, *K*_m_ and *k*_cat_/*K*_m_ were calculated from these measurements (Figure 5 and Supplementary Table S4). For R1, the most significant differences between the two proteins variants could be observed with regard to X5P. The apparent *K*_mX5P_ values of the phosphomimetic AtTKL1_S428D_ at pH 7.2 showed a nearly 2-fold increase compared with the wild-type AtTKL1 that is reflected by a 40% decrease in catalytic efficiency *k*_cat_/*K*_m_ (Figure 5 and Supplementary Table S4). In the case of R2, the most significant differences in the saturation kinetics were observed with F6P as the substrate. In general, both enzymes variants showed a 3–4-fold higher *K*_mF6P_ value at pH 7.2 than at 8.0. In addition, the wild-type enzyme had higher *K*_mF6P_ and *V*_maxF6P_ values compared with the phosphomimetic mutant (Figure 5 and Supplementary Table S4). These changes are reflected by a significant increase of 20–25% in the catalytic efficiency of the phosphomimetic mutant under both pH values (Figure 5).

The differences in the saturation kinetics suggest that phosphorylation at Ser^428^ affects the enzyme activity of TKL depending on specific substrates and pH values, thereby presumably affecting carbon allocation within the metabolic pathways in which TKL is involved. The OPPP is especially important at night or in non-photosynthetic tissues [3] and starts with an oxidative phase that produces X5P and R5P. The non-oxidative phase of the OPPP then progresses with the conversion of X5P and R5P into S7P and G3P by TKL (R1). On the basis of our observations, the phosphomimetic mutant is less effective specifically for X5P under physiological conditions that occur during the night, i.e. pH 7.2. We thus hypothesize that under these conditions phosphorylation of AtTKL1 decreases the affinity of the enzyme to X5P allowing pentose phosphates to be channelled out of the OPPP into other metabolic reactions. For instance, this scenario could happen in response to a cellular need for the synthesis of nucleic acids and other derivatives [3].

The conversion of G3P and F6P into X5P and E4P by TKL (R2) is part of the CBB cycle and therefore of special importance only in photosynthetic tissues during the day. It represents a central decision point since F6P is a precursor of starch synthesis, but is also essential for the regeneration of RBP. It was suggested early on that TKL might play an important role in regulating F6P levels and thereby affect the flux of carbohydrates between the CBB cycle and starch biosynthesis [1]. The remarkably high *K*_mF6P_ value observed in the present study is in agreement with previous estimations that the cellular content of F6P is approximately 5–10-fold higher than the content of R5P [36] and the *in vivo* measurements of spinach TKL that revealed a *K*_mF6P_ value of 3.2 mM [37]. Even with its low affinity, the non-phosphorylated TKL would be able to catalyse normal flow through the CBB cycle. However, as a fast response to external stimuli, a higher demand on E4P would be required for secondary metabolism [4] and processes such as plant defence have been linked to a transient increase in stromal calcium concentration [38]. Upon an increase in calcium concentration, the CBB cycle enzymes FBPase and SBPase (sedoheptulose 1,7-bisphosphatase) undergo inhibition of their activity [39,40]. Together with the withdrawal of E4P this would prevent the regeneration of RBP and the continued operation of the CBB cycle. However, the initial high content of F6P would still allow sufficient production of E4P by TKL for removal out of the CBB cycle. Phosphorylation of TKL would furthermore increase the affinity of TKL for F6P, thereby counteracting the rapid diminishing of the F6P pool under these conditions. Indeed, studies on mutant tobacco plants have shown that small decreases in TKL content caused reduced levels of E4P, leading to photosynthesis inhibition and a significant decrease in aromatic amino acids and soluble phenylpropanoids [41]. This indicates that the small, but significant, differences in saturation kinetics observed for TKL in the present study would be sufficient for regulation of carbon flux.

Taken together, the results suggest that Ser^428^ phosphorylation is not an on/off trigger mechanism for switching metabolic pathways, but rather a fine-tuned mechanism for carbon allocation within different metabolic pathways. This hypothesis is also supported by a recent phosphoproteomic study from orange chromoplasts which showed that the same conserved serine of TKL is phosphorylated at later stages of fruit ripening [30]. On the basis of their finding, the authors suggest that phosphorylation of TKL plays a role in the regulation of the OPPP by affecting anabolism- or glycolysis-related processes [30].

### Conclusion

Regulation of cellular processes often occurs via reversible protein phosphorylation, making it one of the most important post-translational modifications. Nevertheless, it was long considered that TKL is a non-regulated enzyme catalysing a readily reversible reaction. Lately, however, theoretical pathway modelling as well as transgenic approaches have shown that such supposedly non-regulated components may exert a major control on carbon fluxes in the cell [42,43]. On the basis of a network analysis of enzyme activities and metabolic levels in photosynthetic tissue it was suggested that fine-tuning of enzyme activity by allosteric effectors and post-translational modification is important for the regulation of metabolism [44]. Thus phosphorylation of TKL could be one mechanism by which the control of carbon fluxes is achieved. This might also explain the calcium dependency of the phosphorylation reaction. Calcium is an important second messenger that transduces environmental signals into a cellular response and it has been shown that calcium transients and calcium regulation also occur in chloroplasts [15,45,46]. Best described so far is the occurrence of a diurnal rhythm of calcium increase shortly after the transfer of chloroplasts into the dark [45]. This could well be correlated with a calcium-induced change in the activity of the CBB cycle; however, conclusive evidence for the *in vivo* role of TKL calcium-dependent phosphorylation will have to come from further experimental studies that go beyond the scope of the present study, such as identification of the corresponding kinase or complementation of an *Arabidopsis* TKL-knockout mutant with protein variants that are either non-phosphorylatable or mimic permanent phosphorylation.

## Online data

Supplementary data
