# Supplementary material for: Phosphorylation of Arabidopsis transketolase at Ser428 provides a potential paradigm for the metabolic control of chloroplast carbon metabolism
Source: Biochem J. 2014 Feb 14;458(Pt 2):313–22. doi: 10.1042/BJ20130631 (PMC3966265; doi:10.1042/BJ20130631)
Supplement: Supplementary data [file bj4580313add.pdf]

BJ Plant

Biochem. J. (2014) **458**, 313–322 (Printed in Great Britain) doi:10.1042/BJ20130631

online data

SUPPLEMENTARY ONLINE DATA

Phosphorylation of *Arabidopsis* transketolase at Ser[428] provides a potential paradigm for the metabolic control of chloroplast carbon metabolism

Agostinho G. ROCHA*[1], Norbert MEHLMER*, Simon STAEL†[2], Andrea MAIR‡, Nargis PARVIN*, Fatima CHIGRI§, Markus TEIGE†‡ and Ute C. VOTHKNECHT*§[3]

*Department of Biology I, LMU Munich, Großhaderner Str. 2-4, D-82152 Planegg-Martinsried, Germany
†Department of Biochemistry, MFPL, University of Vienna, Dr. Bohr Gasse 9/5, A-1030 Vienna, Austria
‡Department of Molecular Systems Biology (MoSys), University of Vienna, Althanstr. 14, A-1090 Vienna, Austria
§Center for Integrated Protein Science (Munich), Department of Biology, LMU Munich, D-82152 Martinsried, Germany

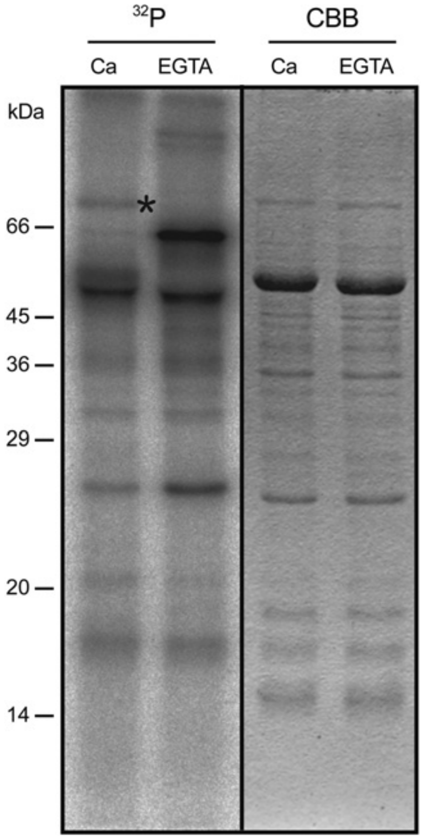

Figure S1 Phosphorylation of stromal proteins from *Arabidopsis*

Analysis of stromal fractions from *Arabidopsis* (10 μg/lane) by SDS/PAGE after phosphorylation assays performed in the presence of either calcium or EGTA. An autoradiogram (left-hand panel) and a Coomassie Blue stain (right-hand panel) are shown. The asterisk indicates phosphorylated TKL. Molecular mass is given on the left-hand side in kDa.

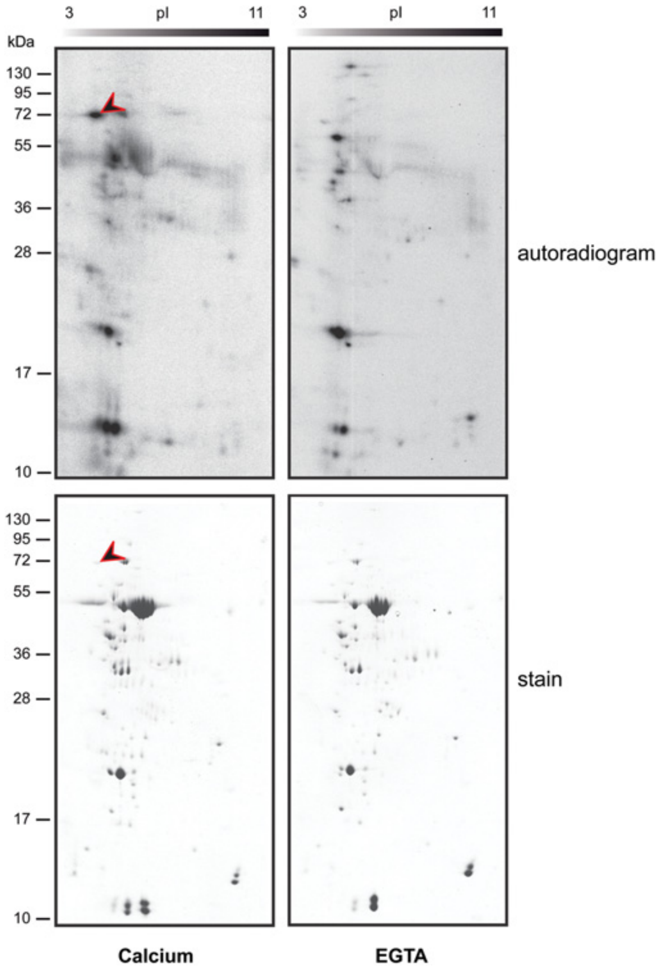

Figure S2 Identification of stromal calcium-dependent phosphoproteins

Autoradiograms (upper panels) and Coomassie Blue-stained gels (lower panels) of stromal proteins from *Arabidopsis* separated by 2D PAGE. Approximately 250 μg of stromal proteins were separated by isoelectric focussing followed by SDS/PAGE, after phosphorylation assays in the presence of either calcium or EGTA. The protein indicated by an arrowhead represents AtTKL1 as subsequently identified by MS/MS. Molecular mass is given on the left-hand side in kDa.

Biochemical Journal www.biochemj.org

[1] Present address: Department of Plant Sciences, University of California, Davis, One Shields Avenue, Davis, CA 95616, U.S.A.
[2] Present address: Department of Plant Systems Biology, VIB, University of Ghent, Technologiepark 927, 9052 Ghent, Belgium.
[3] To whom correspondence should be addressed (email vothknecht@bio.lmu.de).

© 2014 The Author(s)
The author(s) has paid for this article to be freely available under the terms of the Creative Commons Attribution Licence (CC-BY) (http://creativecommons.org/licenses/by/3.0/) which permits unrestricted use, distribution and reproduction in any medium, provided the original work is properly cited.

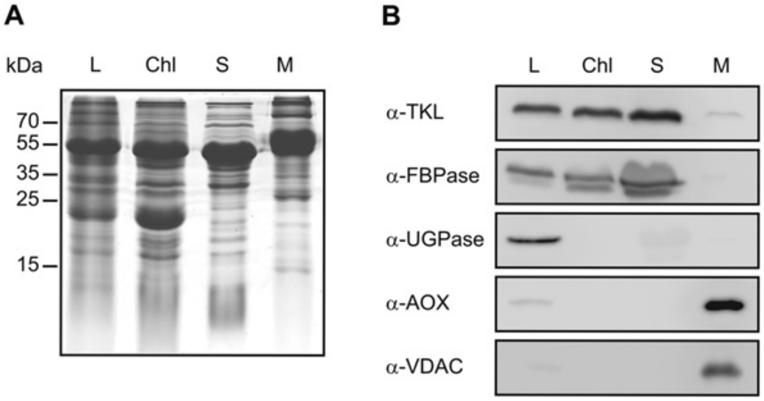

Figure S3 Purity of the stromal extract

To confirm purity of the stromal extract used for phosphorylation assays, Western blot analyses were performed using whole leaf extracts (L), isolated chloroplasts (Chl), stroma (S) and purified mitochondria (M). (**A**) Coomassie Blue staining shows that approximately equal amounts of protein were loaded into each lane. Molecular mass is given on the left-hand side in kDa. (**B**) Immunoblots were performed using antibodies against TKL (α-TKL), stromal FBPase (α-FBPase), cytosolic UDP-glucose pyrophosphorylase (α-UGPase), mitochondrial voltage-dependent anion channel (α-VDAC) and alternative oxidase (α-AOX).

Table S1 Primers used in the present study

fw, forward; rv, reverse.

Name	Sequence
AtTKL-fw	5′-ATAAGCGGCCGCGCTGTTGAGACTGTTGAGCCAACCA-3′
AtTKL-rv	5′-ATAAGGATCCTTAGAAGAATGACTTGGCCG-3′
AtTKLS428D-fw	5′-CACCAGAGGACCCAGGTGATG-3′
AtTKLS428D-rv	5′-ACCTGGGTCCTCTGGTGTGTA-3′
AtTKLS428A-fw	5′-CACCAGAGGCTCCAGGTGATG-3′
AtTKLS428A-rv	5′-ACCTGGAGCCTCTGGTGTGTA-3′
AtTKLYFP-fw	5′-ATAGGGCCCATGGCTTCTACTTCTTCCCTCGCTCTC-3′
AtTKLYFP-rv	5′-ATAGCGGCCGCAGAAGAATGACTTGGCCGCA-3′
CrTKL-fw	5′-ATACATATGCAGACCATGCTGAAGCAGCGCTGCC-3′
CrTKL-rv	5′-ATACTCGAGGTGCTGCAGGGTGGCCTTGG-3′
cpCKII-Apa1	5′-AAGGGCCCATGGCTCAGAAGATCGGTAAATCTATCC-3′
cpCKII-Not1	5′-GCGGCCGCCCTGGCTGCGCGGCGTACGGCTGCTC-3′
E4PDH-fw	5′-ATAGCGGCCGCACCGTACGCGTAGCGATAAATGGCTT-3′
E4PDH-rv	5′-ATAGGATCCTTACCTGAAAGCAACAGTAG-3′

Table S2 Accession numbers used for sequence alignment and residue probability determination

Protein	Organism	EMBL/GenBank® accession number
AtTKL	*Arabidopsis thaliana*	NP_567103
AlTKL	*Arabidopsis lyrata*	XP_002876580
ThTKL	*Thellungiella halophila*	BAJ33959
RcTKL	*Ricinus communis*	XP_002511690
StTKL	*Solanum tuberosum*	CAA90427
PtTKL	*Populus trichocarpa*	ABK92500
NtTKL	*Nicotiana tabacum*	ACF60500
SoTKL	*Spinacia oleracea*	O20250
CaTKL	*Capsicum annuum*	CAA75777
OsTKL	*Oryza sativa*	AAO033154
VvTKL	*Vitis vinifera*	XP_002280760
SbTKL	*Sorghum bicolor*	XP_002437762
ZmTKL	*Zea mays*	ACF88120
PsTKL	*Picea sitchensis*	ACN39962
PpTKL	*Physcomitrella patens*	XP_001769997
SmTKL	*Selaginella moellendorffii*	XP_002991185
OlTKL	*Ostreococcus lucimarinus*	XP_001418785
MpTKL	*Micromonas pusilla*	XP_003061196
VcTKL	*Volvox carteri*	XP_002953691
CrTKL	*Chlamydomonas reinhardtii*	XP_001701881
TeTKL	*Thermosynechococcus elongatus*	NP_682660
PmTKL	*Prochlorococcus marinus*	YP_292380

© 2014 The Author(s)
The author(s) has paid for this article to be freely available under the terms of the Creative Commons Attribution Licence (CC-BY) (http://creativecommons.org/licenses/by/3.0/) which permits unrestricted use, distribution and reproduction in any medium, provided the original work is properly cited.

A

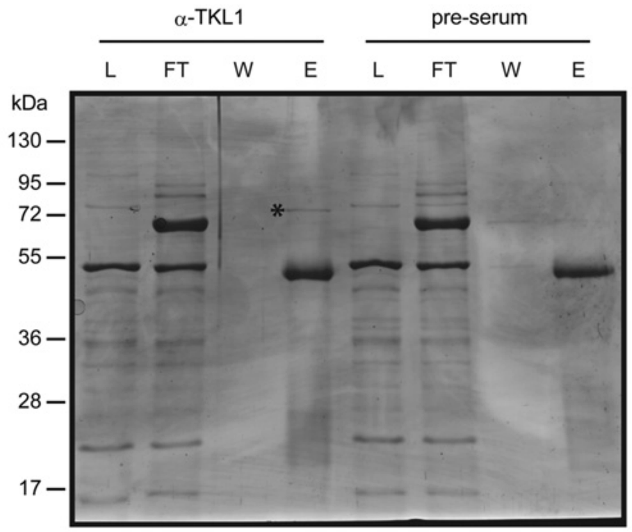

B

```
  1 MASTSSLALS QALLARAISH HGSDQRGSLP AFSGLKSTGS RASASSRRRI AQSMTKNRSL
 61 RPLVRAAAVE AIVTSSDSSL VDKSVNTIRF LAIDAVEKAK SGHPGLPMGC APMSHILYDE
121 VMRYNPKNPY WFNRDRFVLS AGHGCMLLYA LLHLAGYDSV QEEDLKQFRQ WGSKTPGHPE
181 NFETPGIEVT TGPLGQGIAN AVGLALAEKH LAARFNKPDA EVVDHYTYAI LGDGCQMEGI
241 SNEACSLAGH WGLGKLIAFY DDNHISIDGD TEIAFTENVD QRFEALGWHV IWVKNGNTGY
301 DEIRAAIKEA KTVTDKPTLI KVTTTIGYGS PNKANSYSVH GAALGEKEVE ATRNNLGWPY
361 EPFQVPDDVK SHWSRHTPEG ATLESDWSAK FAAYEKKYPE EASELKSIIT GELPAGWEKA
421 LPTYTPESPG DATRNLSQQC LNALAKVVPG FLGGSADLAS SNMTLLKAFG DFQKATPEER
481 NLRFGVREHG MGAICNGIAL HSPGLIPYCA TFFVFTDYMR GAMRISALSE AGVIYVMTHD
541 SIGLGEDGPT HQPIEHIASF RAMPNTLMFR PADGNETAGA YKIAVTKRKT PSILALSRQK
601 LPHLPGTSIE GVEKGGYTIS DDSSGNKPDV ILIGTGSELE IAAQAAEVLR KDGKTVRVVS
661 FVCWELFDEQ SDEYKESVLP SDVSARVSIE AASTFGWGKI VGGKGKSIGI NSFGASAPAP
721 LLYKEFGITV EAVVDAAKSF F
```

Figure S4 Immunopurification of stromal AtTKL1

(**A**) SDS/PAGE analysis of an immunopurification of stromal proteins (8 μg of total protein) after a 'cold' phosphorylation assay using antiserum raised against recombinant AtTKL1 (α-TKL1). Immunopurification with pre-immune serum was used as a control for the specificity of the antibody (pre-serum). A 73 kDa protein (*) was only found in the eluate of the reaction containing anti-TKL1 antiserum and was submitted to phosphopeptide analysis. Molecular mass is given on the left-hand side in kDa. L, load; FT, flow-through; W, wash; E, eluate. (**B**) Sequence coverage assigned to the immunopurified *Arabidopsis* TKL. Grey boxes indicate peptides found by MS/MS. The arrowhead indicates the potential cleavage site for the transit peptide as predicted by similarity to TKL from spinach [1]. An asterisk indicates the identified phosphoserine. The amino acid coverage from the full-length protein is 31.31 % (or 34.42 % when excluding the first 67 amino acids of the targeting sequence).

© 2014 The Author(s)
The author(s) has paid for this article to be freely available under the terms of the Creative Commons Attribution Licence (CC-BY) (http://creativecommons.org/licenses/by/3.0/) which permits unrestricted use, distribution and reproduction in any medium, provided the original work is properly cited.

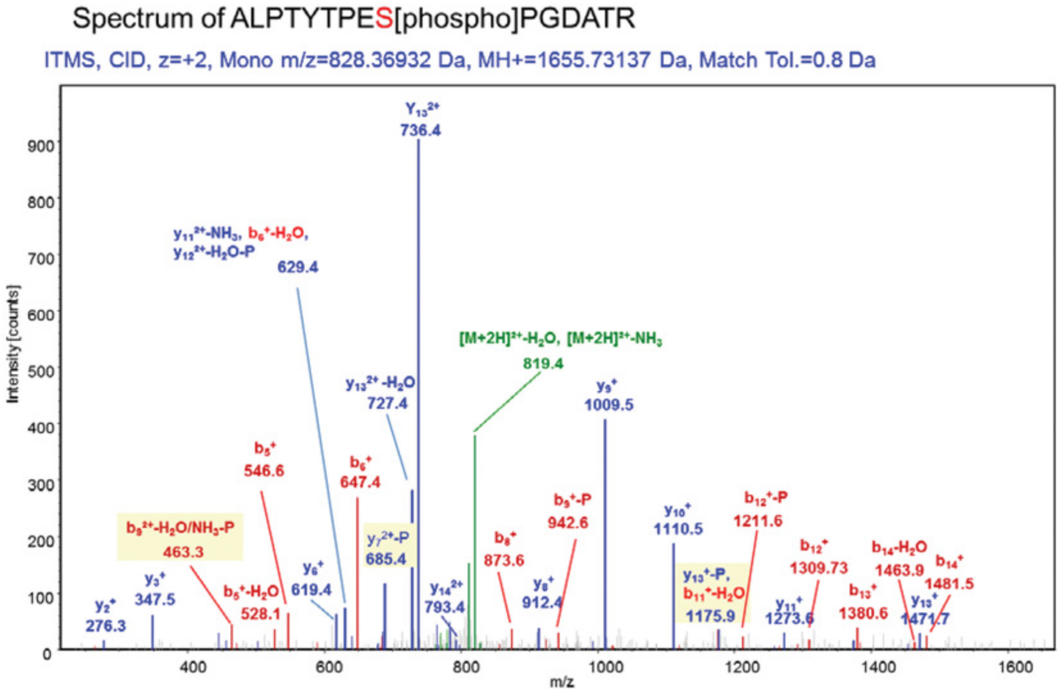

#1	b⁺	b²⁺	Seq.	y⁺	y²⁺	#2
1	72.04	36.52	A			15
2	185.13	93.07	L	1584.69	792.85	14
3	282.18	141.59	P	1471.61	736.31	13
4	383.23	192.12	T	1374.56	687.78	12
5	546.29	273.65	Y	1273.51	637.26	11
6	647.34	324.17	T	1110.45	555.73	10
7	744.39	372.70	P	1009.40	505.20	9
8	873.44	437.22	E	912.35	456.68	8
9	1040.43	520.72	S-Phospho	783.30	392.16	7
10	1137.49	569.25	P	616.30	308.66	6
11	1194.51	597.76	G	519.25	260.13	5
12	1309.53	655.27	D	462.23	231.62	4
13	1380.57	690.79	A	347.20	174.11	3
14	1481.62	741.31	T	276.17	138.59	2
15			R	175.11	88.06	1

Figure S5 Annotated spectrum for the phosphopeptide identified in AtTKL1

Collision-induced dissociation spectrum of the phosphopeptide ALPTYTPES(ph)PGDATR with an XCorr of 3.41, $\Delta m = 0.03$. The annotated spectrum demonstrates phosphorylation at Ser^{428}. B-ions are indicated in red, y-ions in blue, and the precursor-ions after neutral losses (water, ammonia or/and phosphoric acid) are in green.

© 2014 The Author(s)
The author(s) has paid for this article to be freely available under the terms of the Creative Commons Attribution Licence (CC-BY) (http://creativecommons.org/licenses/by/3.0/) which permits unrestricted use, distribution and reproduction in any medium, provided the original work is properly cited.

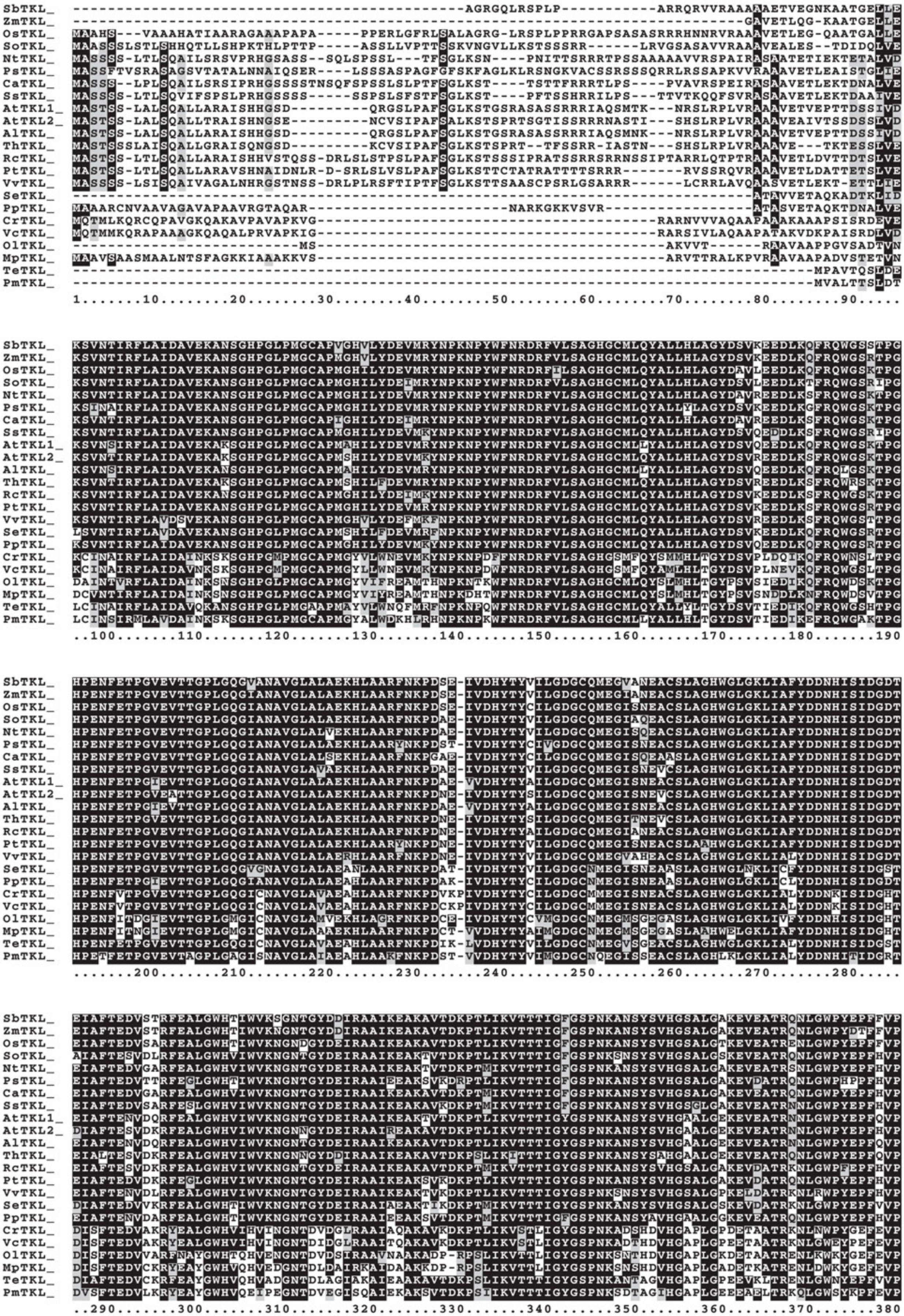

© 2014 The Author(s)
The author(s) has paid for this article to be freely available under the terms of the Creative Commons Attribution Licence (CC-BY) (http://creativecommons.org/licenses/by/3.0/) which permits unrestricted use, distribution and reproduction in any medium, provided the original work is properly cited.

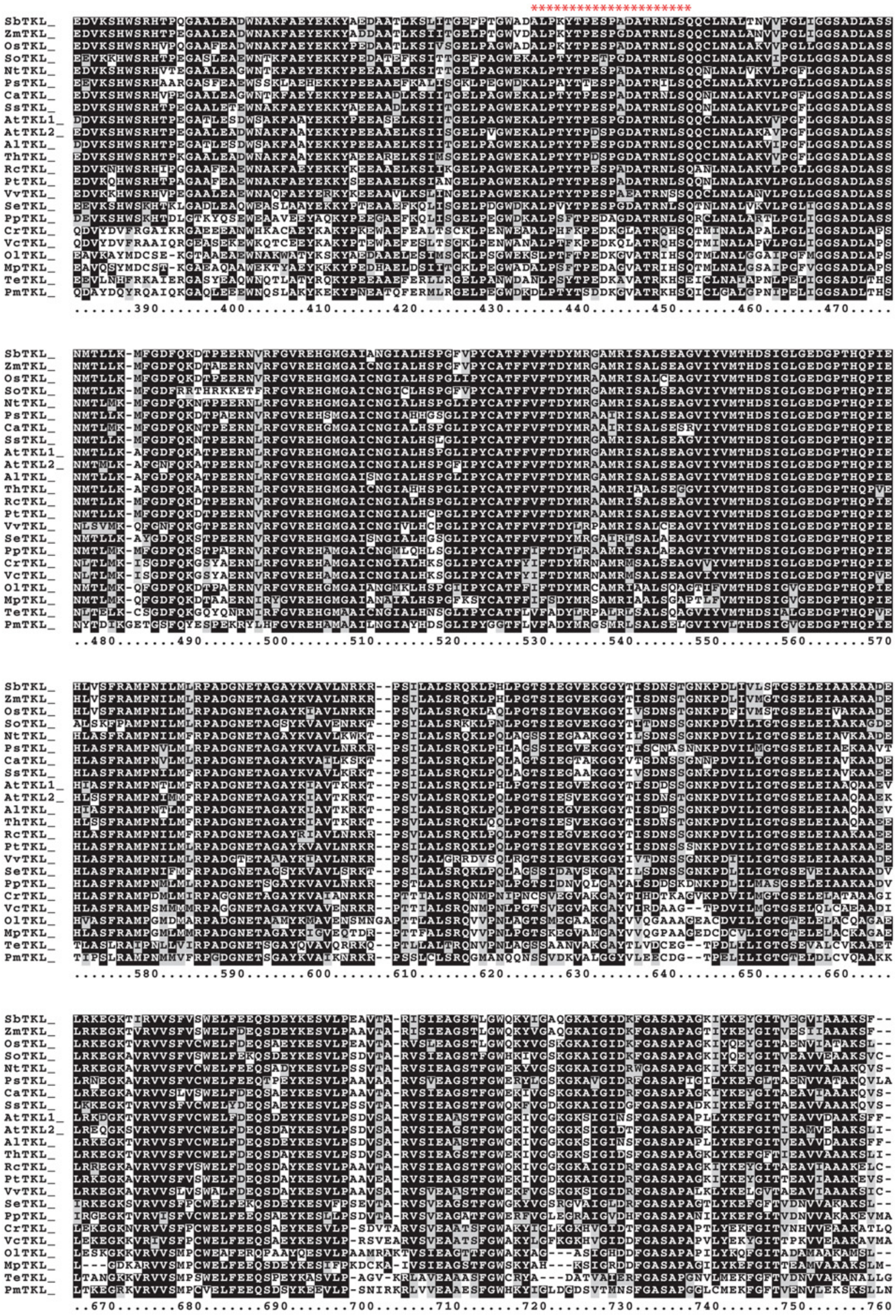

© 2014 The Author(s)
The author(s) has paid for this article to be freely available under the terms of the Creative Commons Attribution Licence (CC-BY) (http://creativecommons.org/licenses/by/3.0/) which permits unrestricted use, distribution and reproduction in any medium, provided the original work is properly cited.

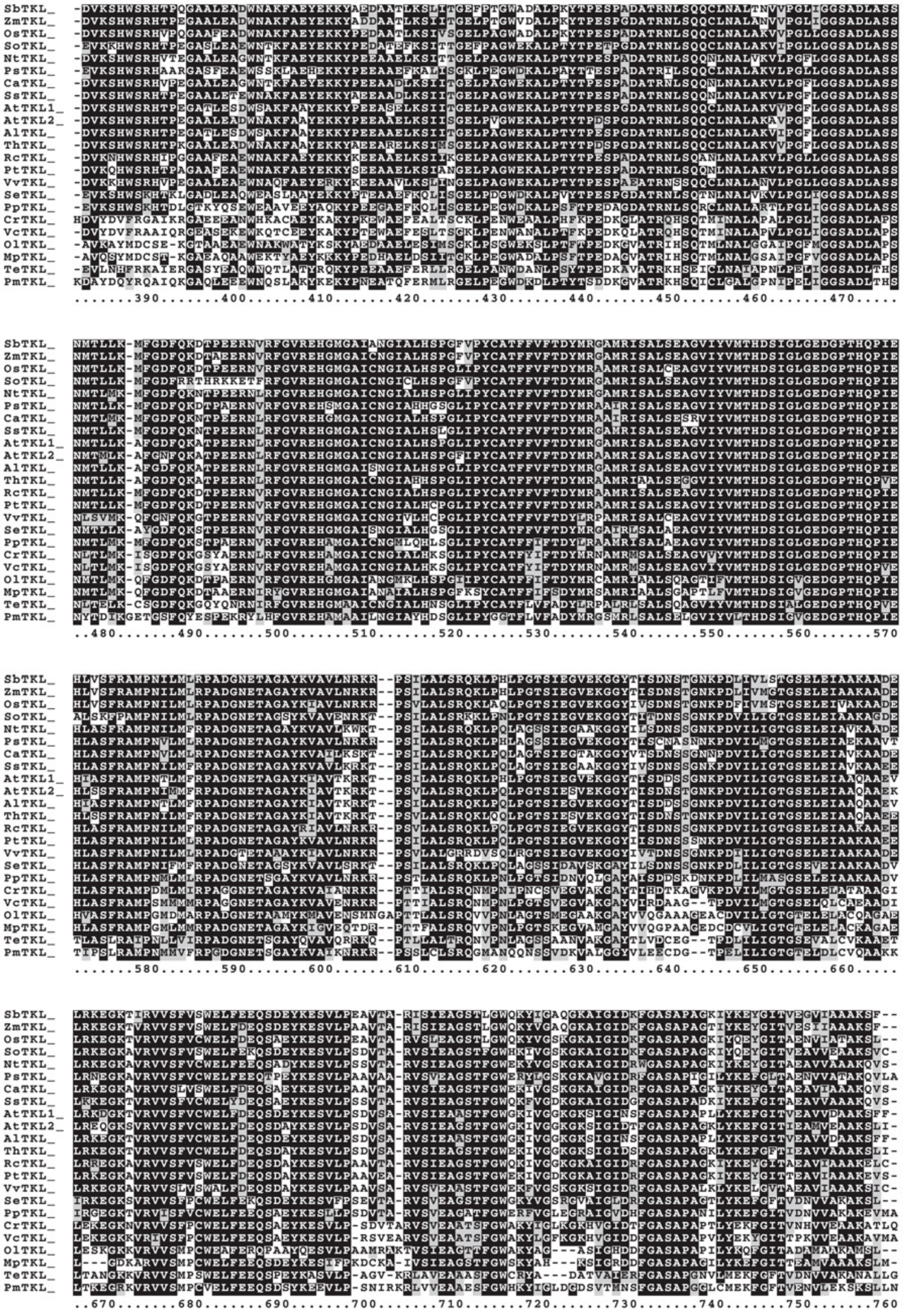

Figure S6 Sequence alignment of TKLs from different photosynthetic organisms

The phosphopeptide identified in AtTKL1 is marked by red asterisks. A complete list of accession numbers is given in Table S2.

© 2014 The Author(s)
The author(s) has paid for this article to be freely available under the terms of the Creative Commons Attribution Licence (CC-BY) (http://creativecommons.org/licenses/by/3.0/) which permits unrestricted use, distribution and reproduction in any medium, provided the original work is properly cited.

Table S3 Sequence coverage and total number of non-redundant peptides assigned to the 72 kDa protein identified as *Arabidopsis* TKL

AT3G60750.1|Symbols:|transketolase, putative|chr3:22454004-22456824 FORWARD. Protein identification probability (100 %), protein percentage of total spectra (0.76 %), total number of spectra (99), number of unique peptides (20) and sequence coverage (26 %).

Peptide sequence	Previous amino acid	Next amino acid	Number of spectra
FAAYEK	Lys	Lys	3
AFGDFQK	Lys	Ala	4
TPSILALSR	Lys	Gln	5
NPYWFNR	Lys	Asp	5
FLAIDAVEK	Arg	Ala	6
YPEEASELK	Lys	Ser	5
KTPSILALSR	Arg	Gln	8
TVTDKPTLIK	Lys	Val	4
NGNTGYDEIR	Lys	Ala	5
ESVLPSDVSAR	Lys	Val	3
KYPEEASELK	Lys	Ser	9
VTTTIGYGSPNK	Lys	Ala	8
NLSQQCLNALAK	Arg	Val	6
SIITGELPAGWEK	Lys	Ala	2
ANSYSVHGAALGEK	Lys	Glu	3
LPHLPGTSIEGVEK	Lys	Gly	4
ALPTYTPESPGDATR	Lys	Asn	10
HTPEGATLESDWSAK	Arg	Phe	3
QKLPHLPGTSIEGVEK	Arg	Gly	4
SIGINSFGASAPAPLLYK	Lys	Glu	2
Total			99

Table S4 Substrate saturation kinetics of heterologously expressed wild-type (wt) or phosphomimetic (S428D) AtTLK1

Enzymatic reactions were measured either with X5P and R5P or with F6P and G3P at pH 7.2 and 8.0. Kinetic parameters were calculated for all sugars and each value represents the mean ± S.D. for four to five independent determinations. Significance of the parameters was calculated using a standard Student's *t* test. NS, not significant; WT, wild-type.

Sugar	Parameter	pH 7.2			pH 8.0		
		WT	S428D	*P*	WT	S428D	*P*
X5P	V_{max} (μmol/min per mg)	14.6 ± 0.4	15.0 ± 0.4	NS	13.2 ± 0.4	13.5 ± 0.4	NS
	K_m (μM)	170.6 ± 15.7	260.2 ± 16.9	<0.01	117.1 ± 11.7	104.9 ± 17.6	NS
	K_{cat}/K_m ($M^{-1}\cdot s^{-1}$)	$2.1\times10^5 \pm 1.7\times10^4$	$1.4\times10^5 \pm 5.4\times10^3$	<0.01	$2.8\times10^5 \pm 2.0\times10^4$	$3.3\times10^5 \pm 2.0\times10^4$	NS
R5P	V_{max} (μmol/min per mg)	18.1 ± 0.3	17.6 ± 0.2	NS	20.4 ± 0.3	20.1 ± 0.3	NS
	K_m (μM)	478.8 ± 6.7	499.3 ± 11.7	NS	1217.0 ± 24.2	1355.0 ± 42.4	<0.05
	K_{cat}/K_m ($M^{-1}\cdot s^{-1}$)	$9.1\times10^4 \pm 1.1\times10^3$	$8.6\times10^4 \pm 1.3\times10^3$	<0.05	$4.1\times10^4 \pm 1.3\times10^3$	$3.6\times10^4 \pm 1.1\times10^3$	<0.05
G3P	V_{max} (μmol/min per mg)	0.7 ± 0.0	0.8 ± 0.0	<0.05	1.7 ± 0.4	1.9 ± 0.1	NS
	K_m (μM)	127.0 ± 6.5	161.4 ± 11.4	<0.05	910.2 ± 425.4	891.9 ± 85.6	NS
	K_{cat}/K_m ($M^{-1}\cdot s^{-1}$)	720 ± 37	620 ± 32	NS	320 ± 72	260 ± 19	NS
F6P	V_{max} (μmol/min per mg)	4.0 ± 0.1	2.4 ± 0.0	<0.001	5.5 ± 0.0	4.1 ± 0.0	<0.01
	K_m (μM)	23.1 ± 0.6	11.4 ± 0.2	<0.001	7.4 ± 0.1	4.1 ± 0.2	<0.01
	K_{cat}/K_m ($M^{-1}\cdot s^{-1}$)	14 ± 0.2	17×10 ± 0.1	<0.001	61 ± 0.9	80 ± 3.3	<0.01

REFERENCES

1 Teige, M., Melzer, M. and Suss, K. H. (1998) Purification, properties and *in situ* localization of the amphibolic enzymes D-ribulose 5-phosphate 3-epimerase and transketolase from spinach chloroplasts. Eur. J. Biochem. **252**, 237–244

Received 8 May 2013/3 December 2013; accepted 13 December 2013
Published as BJ Immediate Publication 13 December 2013, doi:10.1042/BJ20130631

© 2014 The Author(s)
The author(s) has paid for this article to be freely available under the terms of the Creative Commons Attribution Licence (CC-BY) (http://creativecommons.org/licenses/by/3.0/) which permits unrestricted use, distribution and reproduction in any medium, provided the original work is properly cited.
